# Emerging Technologies for Assessing Physical Activity Behaviors in Space and Time

**DOI:** 10.3389/fpubh.2014.00002

**Published:** 2014-01-28

**Authors:** Philip M. Hurvitz, Anne Vernez Moudon, Bumjoon Kang, Brian E. Saelens, Glen E. Duncan

**Affiliations:** ^1^Urban Form Laboratory, Department of Urban Design and Planning, University of Washington, Seattle, WA, USA; ^2^Department of Urban and Regional Planning, State University of New York, Buffalo, NY, USA; ^3^Seattle Children’s Research Institute, Seattle, WA, USA; ^4^Department of Pediatrics, University of Washington, Seattle, WA, USA; ^5^Nutritional Sciences Program, Department of Epidemiology, University of Washington, Seattle, WA, USA

**Keywords:** accelerometry, behavior, environment, geographic information systems, global positioning systems, physical activity

## Abstract

Precise measurement of physical activity is important for health research, providing a better understanding of activity location, type, duration, and intensity. This article describes a novel suite of tools to measure and analyze physical activity behaviors in spatial epidemiology research. We use individual-level, high-resolution, objective data collected in a space-time framework to investigate built and social environment influences on activity. First, we collect data with accelerometers, global positioning system units, and smartphone-based digital travel and photo diaries to overcome many limitations inherent in self-reported data. Behaviors are measured continuously over the full spectrum of environmental exposures in daily life, instead of focusing exclusively on the home neighborhood. Second, data streams are integrated using common timestamps into a single data structure, the “LifeLog.” A graphic interface tool, “LifeLog View,” enables simultaneous visualization of all LifeLog data streams. Finally, we use geographic information system SmartMap rasters to measure spatially continuous environmental variables to capture exposures at the same spatial and temporal scale as in the LifeLog. These technologies enable precise measurement of behaviors in their spatial and temporal settings but also generate very large datasets; we discuss current limitations and promising methods for processing and analyzing such large datasets. Finally, we provide applications of these methods in spatially oriented research, including a natural experiment to evaluate the effects of new transportation infrastructure on activity levels, and a study of neighborhood environmental effects on activity using twins as quasi-causal controls to overcome self-selection and reverse causation problems. In summary, the integrative characteristics of large datasets contained in LifeLogs and SmartMaps hold great promise for advancing spatial epidemiologic research to promote healthy behaviors.

## Introduction

The health benefits of regular physical activity are well established, including weight control, improved cardiorespiratory fitness, and reduced risk of developing chronic diseases such as type 2 diabetes, cardiovascular disease, and some forms of cancer (1–5). Despite these recognized benefits, most people in the U.S. do not engage in physical activity at levels consistent with recommendations for health benefits (6, 7). Precise measurement of physical activity behaviors, including type, amount, context, and place, is essential for increasing physical activity at the population level because it enables a better understanding of where, when, and how much activity is or is not occurring. Emerging technologies are increasingly being used to improve the precision and accuracy of objective physical activity measurement and to enable detailed examinations of where and when physical activity behaviors actually occur. While these technologies greatly advance the field of physical activity research, they also present entirely new methodological challenges. For example, the large amount of data produced when multiple participants wear accelerometers and global positioning system (GPS) devices over the course of several days generates new requirements for data structure and processing. A typical 7-day period of monitoring using a 1-min collection window yields over 1000 observations per person; in one recent study, ~88% of these data points were dropped because of computational incapability with such a large data size (8). Because many studies collect refined data at high temporal resolution, new tools that can deal with such large data sets are necessary. In addition, the multiple activity, location, and environment data streams need to be integrated into a comprehensive structure that permits combined analyses of behaviors in time and space. Although increasing numbers of studies are using these integrated technologies, there is little technical guidance for researchers who want to use these methods in their studies.

This paper introduces a novel suite of data collection instruments, data management tools, and analytic methods to measure and analyze activity behaviors that have broad applications in spatial epidemiology. We focus on individual-level, high-resolution, objective data on activity, location, and environment. First, we describe and assess a range of instruments used to capture physical activity and its location over the course of daily life. These instruments include accelerometers, GPS data loggers, and travel diaries. Second, we present a set of tools, which were created to manage the large data sets generated by accelerometry and GPS. The first data management tool is the “LifeLog,” which combines accelerometry- and diary-based activity and GPS-derived location data streams into a single temporal data structure using a common timestamp for data linkage. The LifeLog is in turn complemented by the “LifeLog View,” a graphic display interface tool that enables simultaneous visualization of activity and location data streams. These tools yield a common spatial–temporal data structure for activity and location that is also necessary to investigate high-resolution built and social–environmental influences on physical activity behaviors. Third, we seek to bypass the limitations of past research, which only considered the influence of the home environment or “neighborhood” on behavior; instead, we measure physical activity across the full spectrum of exposures encountered in daily life. To do so, we have developed a new approach to capture the attributes of the built and social environments at the many locations generated by GPS data. We introduce SmartMaps, a tool for environmental data management. SmartMaps are rasterized or grid-based surfaces, which provide spatially continuous values of environmental attributes. The fine-grained grid-based measures of environment calculated by the SmartMaps serve to capture exposures with the same spatial and temporal resolution as that obtained by accelerometry and GPS.

The emerging technologies embodied in the set of instruments and management tools presented here promise to precisely measure and analyze physical activity behaviors in various settings over the full spatial–temporal continuum. They have been used in a few studies to date, two of which are described in this article, including a natural experiment to evaluate the effects of new transportation infrastructure on physical activity levels and a neighborhood-effects study that features twins as quasi-causal controls to overcome self-selection and reverse causation problems. Finally, we discuss both the great potential and limitations of the tools and methods presented and suggest future studies that would further advance spatial epidemiologic research.

## Materials and Methods

### Activity behavior and location data collection instruments

Our instruments include accelerometers, GPS devices, smartphones, as well as both paper and digital travel diaries.

#### Accelerometer

We use accelerometers to assess physical activity patterns over time, configuring them for various purposes. In one study, accelerometers were configured to record at minimum acceleration in one-axis (orthogonal to earth surface) while in other studies, the configuration included three-axis accelerometry, steps, incline, and ambient light levels. Measurement epochs for the accelerometer were set to match GPS recording intervals, ranging from 15 s to 1 min.

We use standard off-the-shelf accelerometers such as the ActiGraph GT1M and GT3X models for the objective measurement of physical activity. As one explicit example, accelerometry data were downloaded using ActiLife software (v3.4.0, ActiGraph LLC., Pensacola, FL, USA) and exported as comma-separated value (CSV) text files containing fields for timestamps and the various sensor data streams (i.e., axis counts), text files containing an informational header, including starting timestamp, epoch duration, and epoch accumulated values (“DAT” format), or native structured query language (SQL) format (“AGD” files). Text files were imported into a PostgreSQL (9) database, either directly from CSV files or using scripts within the statistical program R (10) that pre-processed the DAT or AGD data into tables containing one record per epoch. Accessing data using R to connect to the SQLite AGD files allowed an automated approach for processing multiple files, rather than requiring a technician to export individual CSV or DAT files using ActiLife software on a per-subject basis.

Our group currently uses the GT3X+ model and the latest version of ActiLife software (v6.8.1). One major innovation is that the latest model now collects and stores raw accelerations, so that epoch duration can be chosen after data collection at the time of data export. This allows accelerometry data to be matched to the data collection interval of any other recording device. We use accelerometry data as the base table to enforce the temporal sequence of the merged dataset containing input from multiple instruments. We adopt this approach because, once the accelerometer starts collecting data, it continues to record regularly until the unit runs out of power, reaches the configured “stop recording” date/time, or malfunctions, whereas other data collection devices may not record regularly or continuously. The unit does not permit any participant input (e.g., it has no on/off switch or other end-user configuration options) or rely on any other input after starting, which reduces participant burden and avoids potential user error. Accelerometry activity count data were processed to yield time-stamped intensity levels for physical activity using commonly accepted thresholds for differentiating activity levels (11) and to examine records for the number of complete wearing days (7).

Using accelerometer count thresholds for estimating physical activity intensity is problematic because these *a priori* defined thresholds do not necessarily take into account individual-level biometric differences, such as variation in body size or aerobic fitness level, and they do not allow for the estimation of physical activity type or context. Promising work is being conducted using a variety of novel methods, including quadratic discriminant analysis and hidden Markov models (HMM) to recognize common physical activities (12), as well as machine-learning algorithms that exploit artificial neural networks (13, 14). Our own work with these novel methods is described briefly in Section “Multi-Sensor Board” below. Indeed, the measures proposed herein may be used as validation strategies for such algorithms. The “packaging” of these algorithms within easily used software will help researchers who are measuring activity levels with accelerometry but who have little experience in software development.

#### Global positioning systems

Our ongoing studies use GPS data loggers to record geospatial locations so that we can assess the spatial and temporal characteristics of travel and “dwell” patterns (e.g., sojourn at a home or work location), including characteristics of specific travel modes. We explain how we conflate the GPS and accelerometry data below in Section “Data Integration, Management, and Visualization Tools.”

We currently use off-the-shelf models such as the GlobalSat (New Taipei City, Taiwan) DG-100 that is equipped with the SiRF Star III/LP 20-channel chipset, and the Qstarz (Taipei, Taiwan) BT-1000XT that contains the MTK 51-channel chipset. Both models feature solid-state memory and rechargeable batteries that allow at least one full day of measurement per charge and up to several weeks of data storage, depending on recording interval and data type.

The DG-100 manual states its accuracy as 10 m, whereas the stated accuracy of the BG-1000XT is 3 m. The DG-100 can record a maximum of only 5 values per record, including position, timestamp, speed, and altitude, whereas the BG-1000XT can record up to 19 values, including the previous 4, as well as data quality variables such as dilution of precision, number of satellites used in the fix, satellite position, and signal-to-noise ratio.

We collect data in binary format and export them as CSV files, with one record per logging interval during which a fix was determined (at least four satellites in view and a horizontal dilution of precision less than eight). Consumer-level GPS units such as the DG-100 and BG-1000XT can be configured to log at regular intervals, such as 15 s, but they begin recording as soon as a fix is obtained (rather than at a time evenly divisible by 15 s) and store the next record after the configured interval has elapsed.

The GPS data are processed and stored in a PostgreSQL database enabled with PostGIS, the spatial data storage and analysis extension (15). Longitude and latitude coordinates are used to generate spatial point features for mapping and spatial analysis. Unlike the data structure obtained from accelerometry, GPS data frequently contain large intervals without data, caused by signal reception failure due to such factors as obstruction of line-of-sight with GPS satellites, powering down during recharging, or cold starts (delays between starting up and acquiring a satellite signal).

#### Multi-sensor board

Our team also uses a multi-modal sensor known as the multi-sensor board (MSB), which was developed by researchers at the University of Washington in collaboration with Seattle Intel Labs. This is a pager-sized device worn clipped to a belt (16). It offers a suite of features, including multiple sensing (three-axis accelerometry, barometric pressure, humidity, temperature, light, audio, and GPS), data storage, communication, and local computation capabilities. Rather than outfitting study participants with several different (separate) devices, the MSB records multiple sensor data streams simultaneously. Its functionality yields notable benefits; participants need to wear and recharge only one device, and each variable is recorded in a single binary file, rather than in several files that need management and conflation after download.

As an experimental device, the MSB has various drawbacks, such as limited data storage, limited battery life, and the need for expert staff to configure the devices and to download and transform the multiple data streams. Despite these limitations, it enabled us to develop sophisticated machine-learning algorithms to quantify physical activity types and estimate corresponding energy expenditures that were subsequently validated in laboratory and field experiments (17, 18).

The advantages in using single devices that have multiple sensors and capabilities – such as the MSB and mobile phones – make them an important area for further development and eventual deployment. Although we are currently using stand-alone accelerometers, GPS devices, and mobile phones in many of our research projects, we are benefiting from our previous validation work and using our machine-learning algorithms to obtain richer data on activity amount (i.e., specific activity types and associated energy expenditures) than can be provided by accelerometry and GPS alone.

#### Travel diary instruments

Our research agenda is driven by objective data sources. However, we have found that an important set of behavioral data is not yet available solely through objective measurement. Data for behavioral variables or characteristics such as activity purpose, visited place names and addresses, and certain modes of travel between places cannot, in general, be collected without some user input. Other activities that are difficult to determine, such as walking or jogging on a treadmill or using a stationary bicycle or elliptical machine, would likely require substantial work to be identified solely from objective data.

Other behavioral variables are impractical or impossible to measure with existing instruments. For example, although some devices, such as the ActiGraph GT3X+, are water resistant, most current electronic devices, including GPS units, must be removed during bathing or swimming, preventing the recording of such activities. In addition, objectively sensing behaviors such as eating and food shopping would require the development of new instruments and data processing methods. Given the lack of such instruments, but also the need for obesity-related research to estimate where and when all exercise, travel, and food-related behaviors occur, we created several travel and food diary instruments. For each visited place, key variables include place name, address, arrival and departure time, arriving travel mode, and activity or purpose.

##### Paper version of travel diary

**Figure 1:**
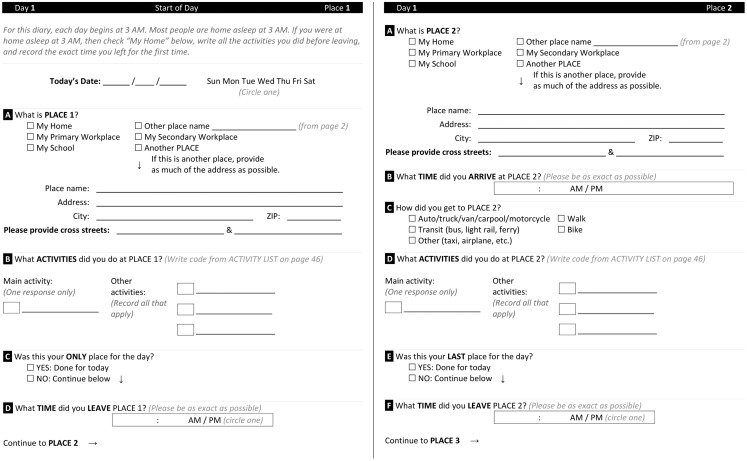
**Paper travel diary for two places in a single travel day**.

We originally created paper booklets with enough blank pages to account for 14 places per day, with extra pages for additional places and days. Participants logged place names, addresses, times of arrival and departure, activities at each place, and mode of travel from place to place. An example of a paper travel diary that we have used in our research is shown in Figure 1.

**Figure 2:**
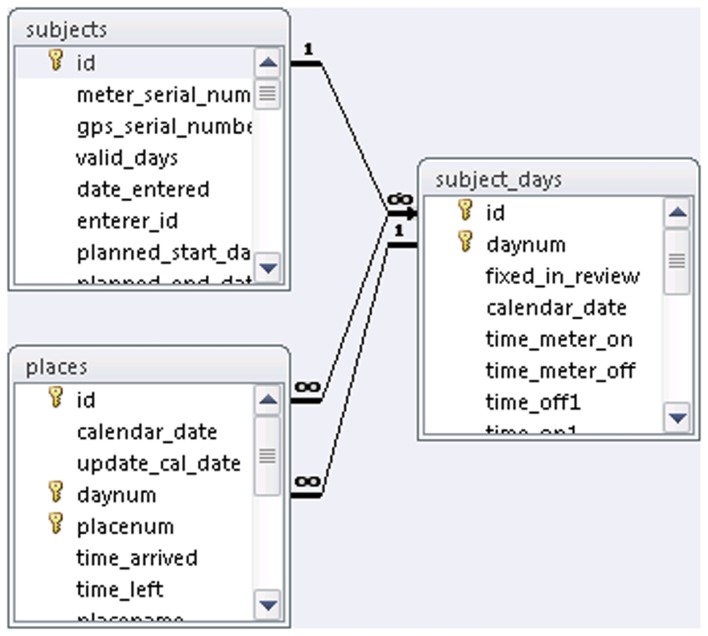
**Travel log database schema for data entry in Microsoft Access**.

We wrote a custom Microsoft access database (MDB) application to facilitate transcription from the paper diary to a digital format. This database automatically links participants, recording days, and place records (see Figure 2). Each participant’s data are stored in a hierarchy identified and linked by participant ID, day number, and record number. The application uses two separate MDB files, one containing the data and the other with the forms and visual basic for applications (VBA) code. The “code” database uses the Linked Data Manager in Access to display the data tables, which are actually stored in the separate “data” MDB file. This structure permits updates to the code database without the need for copying data tables.

**Figure 3:**
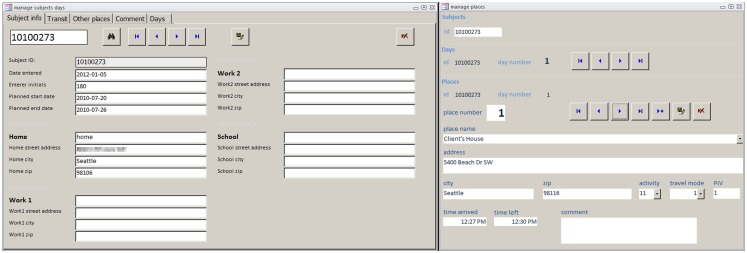
**Travel log database entry forms**. Upper panel: common places; lower panel: a single place record.

**Figure 4:**
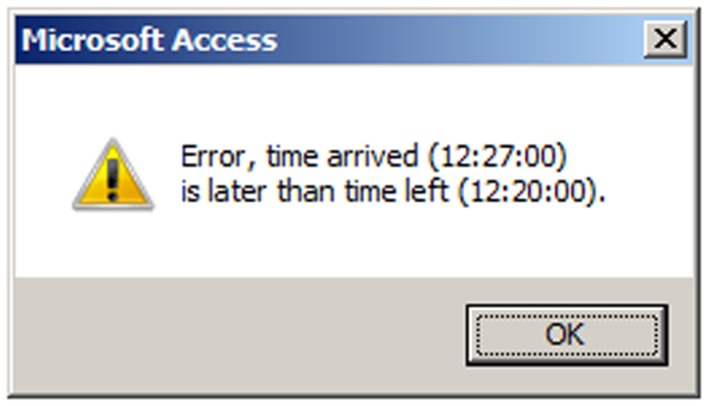
**System generated warning for illogical timestamps**.

The code database contains forms that allow easy navigation among records for participants, participant days, and places as illustrated in Figure 3. The data entry form for place data contains VBA code for simple error checking of intra-place records. For example, if a place record has the “time arrived” later than the “time left,” a warning is generated similar to that shown in Figure 4. The code database also contains queries that display inter-place error checks (e.g., if the “time left” for place 1 is later than the “time arrived” at place 2), allowing data entry staff to review and correct inter-place sequencing errors.

##### Digital versions of travel diary

Although our paper diary was easy to create, edit, and administer, the tedious transcription process, which used the Access database, was vulnerable to errors. Quality control can expose errors (e.g., a.m. and p.m. substitutions, transposed numerals, missed records), but each potential error required manual review to determine whether it originated in the participant’s initial recording or in the transcription process. Furthermore, because data entry and data processing were performed by different study staff, interpreting errors often required communication between research staff (at the same or across sites) and retrieving paper documents from archives.

To avoid the logistical problems associated with paper diaries, we wrote two separate travel diary applications for the Android smartphone platform. Data collected by smartphone do not require transcription, and error-checking can be built into the application, with immediate feedback asking the user to correct impossible entries (e.g., leaving a place before arriving at that place). This approach minimizes or obviates the need for temporal error checking in post-processing.

*My footprints*. We initially used the *Footprints* application for HTC (New Taipei City, Taiwan) Android phones to record the time and location of specific activities (exercise, eating, and food shopping), to encourage participants to create diary records at the time specific activities occurred. This application works by enabling the user to take a digital photo that is automatically tagged with timestamp and location by the phone’s locational sensor and then manually tagged with other user-entered variables. However, *Footprints* offered few options for configuration. For example, the values for the “activity” variable were pre-populated and not editable, so that it was impossible to record various activities of interest (e.g., food shopping) without resorting to the open-ended “comment” variable.

**Figure 5:**
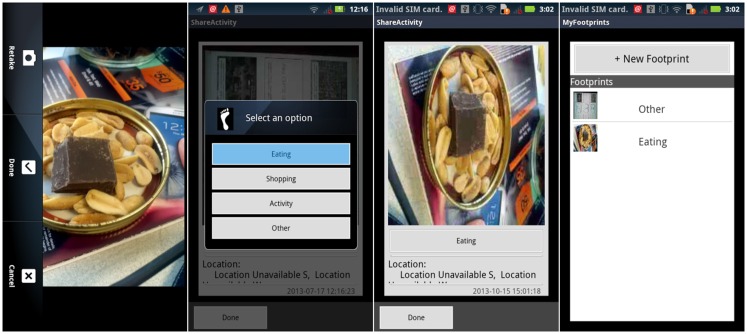
**Examples of *My Footprints* screen captures**. Far left panel: capturing an image with the mobile phone camera; center-left panel: tagging the picture as “eating;” center-right panel: review of image and tag; far right panel: overview of recorded activities.

Instead, we wrote a separate application named *My Footprints* to be more directly useful in our research. A record in *My Footprints* is illustrated in Figure 5, which includes the digital photo filename, an automatically generated timestamp, spatial coordinates for the location where the photo was taken (although not shown in this image capture), and one of four different activities. Data collected with *My Footprints* can be directly transferred from a smartphone to the PostgreSQL database.

*Smartphone-based travel diary*. We also pilot-tested the *Memento* database application for Android phones as a place-based travel diary. This highly flexible and configurable app was able to store all our required fields. However, when data were exported, place records appeared in a seemingly random order, rather than in the order in which they were visited. This is problematic because place sequencing is a basic functional requirement for our research questions. Proper sequencing of places is not an issue for paper travel logs. Rows in the log are numbered sequentially, so we can assume that participants record places in the correct order, and that place numbers are transcribed accordingly.

**Figure 6:**
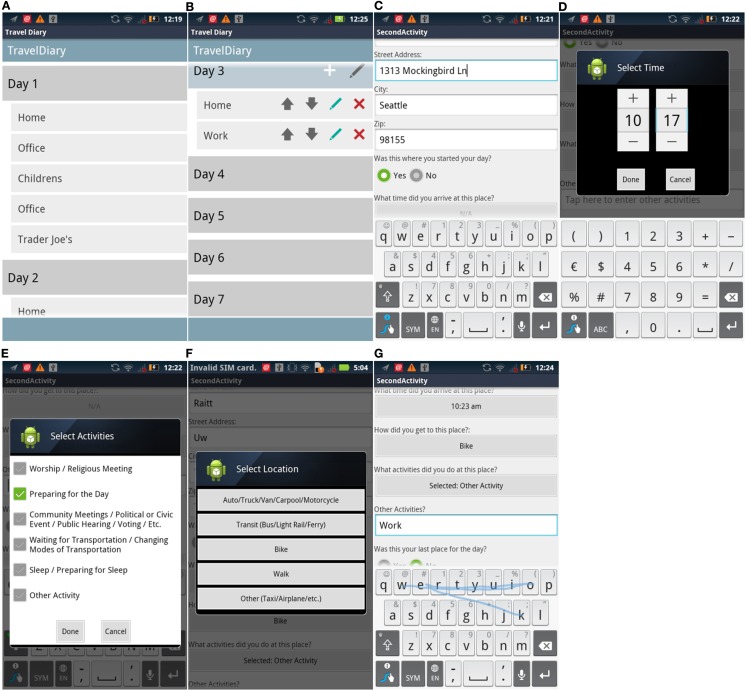
**Travel Diary screen captures**. From left to right, top to bottom: **(A)** layout of place records; **(B)** editing mode for places within a day; **(C)** address place data entry; **(D)** assigning timestamps; **(E)** selecting activity for a place; **(F)** selecting predefined location; **(G)** entering open-ended activity.

Although we were unable to find an effective way to correct the sequencing problem in *Memento*, we created a second Android app simply called *Travel Diary*. This application allowed recording and reordering of days and places (shown in Figures 6A,B), place name, address, time arrived and left (Figures 6C–E), and travel mode and activity (Figures 6F,G).

##### Travel diary processing

Whether paper or digital, the travel diary uses place as the unit of measure. Instead of being stored as data, trips are created as the temporal interstices between places, and generated for each successive pair of place records. A set of R scripts converts the travel diary data into format-standardized CSV files. These files are uploaded to the PostgreSQL database for integration with the GPS and accelerometry data as described in Section “Data Integration, Management, and Visualization Tools” below.

### Built environment measures using SmartMaps

Using GPS to capture location information generates very large amounts of data. We needed a novel approach to effectively measure built environment characteristics at any or all GPS-derived locations recorded from participants. Previous approaches have used spatial buffers around participants’ geocoded residential addresses to extract and summarize geographic information system (GIS) data within the local neighborhood, storing values as individual-level variables (19). However, this point-centric measurement approach requires a substantial amount of data processing for each location. It is also too computationally intensive to be practical for large GPS datasets collected under participant free-roaming conditions.

To address these issues, rather than performing point-centric measures of the built environment at all GPS locations, we created SmartMaps for each built environment attribute of interest. SmartMaps are raster layers (20) – that is spatially continuous surfaces of grid cells – which enable efficient measurements at any number of locations within a study area. The point value at each SmartMap cell represents a summary of the local neighborhood value around that cell. SmartMaps provide the same built environment values as those generated by the traditional buffer method. However, instead of recording neighborhood summaries at specific, predefined point locations, SmartMaps do so for every cell, continuously across space, thereby enabling measures at any location in the study area.

SmartMaps are created by focal raster processing. The area of interest (in our case, King County, WA, USA) is represented as a grid of 30 m× 30 m cells, a resolution that has been shown to represent urban and suburban parcels with sufficient spatial fidelity (21). Each focal cell in the grid is processed independently by using the ArcGIS Spatial Analyst Extension. The software performs prescribed calculations for the neighborhood around the focal cell, places the resulting value on that cell, and then moves on to the next cell, repeating the process until values are calculated for all cells. In our current studies, we use a radius of 833 m to represent the focal “neighborhood,” corresponding to the distance that can be walked in 10 min. For example, to calculate a SmartMap of the count of residential units within 833 m of a specified grid cell, parcels are first converted into a raster grid in which cell values represent the fraction of residential units within the cell (e.g., a 9000 m^2^ parcel containing 20 residential units yields 10 cells with a value of 2 units per cell). The process then sums the values of all cells within each focal buffer to represent the number of residential units in that focal cell’s neighborhood. SmartMap cell values can then be extracted for GPS points by using the ArcGIS Surface Spot analytical method.

For our studies, we have generated SmartMaps that characterize elements of the built environment. These SmartMaps cover domains that past research has associated with physical activity and obesity. For example, neighborhood composition could be represented by counts or densities of employes and residential units (22, 23). Utilitarian or recreational destinations could be captured as counts or densities of supermarkets, fast food outlets, traditional restaurants, coffee shops, fitness facilities (24, 25), or by count of parks, etc. (8, 26). Transportation infrastructure is measured as density of intersections, streets, urban trails, etc. (23, 27, 28). Traffic conditions are represented by estimated traffic volumes (23) and bus ridership as a measure of transportation system load (29).

**Figure 7:**
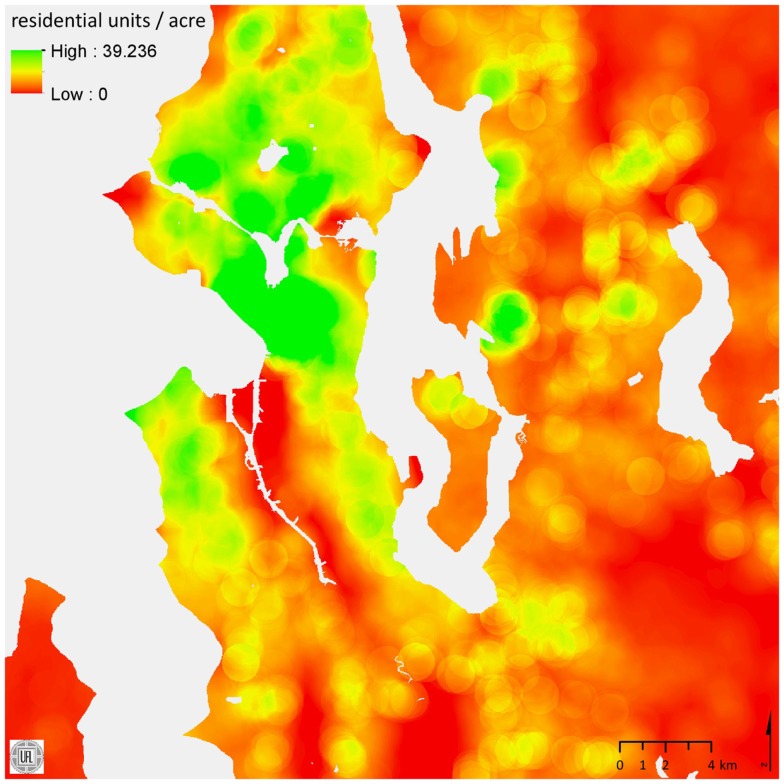
**A SmartMap of residential unit density**. Values are count of residential units per acre within 833 m of each raster cell.

Each one of our SmartMaps of the 5975 km^2^ area of King County contains more than 6.8 million 900 m^2^ (30 m× 30 m) cells, with each cell providing values for the various built environment variables in the associated neighborhood. A SmartMap of the count of residential units within 833 m of each cell is shown in Figure 7.

Using SmartMaps to obtain environmental measures for point locations is considerably more efficient than performing a series of point-centric buffer analyses. For the 3.8 million GPS locations that we collected in one study, less than 1 h per SmartMap was required to extract built environment data in the form of summaries of each 833 m neighborhood (30). Although creating SmartMaps for an area requires substantial effort, the resulting rasters can readily be used in any subsequent study to analyze point measures of the environment within a specified area. SmartMaps are essential for the growing number of studies that use geolocation technologies to track individual movements. Ideally, urban areas would develop sets of SmartMaps for use by multiple agencies or research entities that examine the effects of built environment on behavior. Similar efforts have already been made in fields such as meteorology and noise mitigation (31). Furthermore, SmartMaps can be archived from data sources measured at different points in time for use in longitudinal studies.

### Data integration, management, and visualization tools

We created tools to manage and integrate the massive data streams collected by devices in order to examine relationships between exposures and behaviors. These include LifeLogs and rasterized SmartMaps.

#### LifeLogs

**Figure 8:**
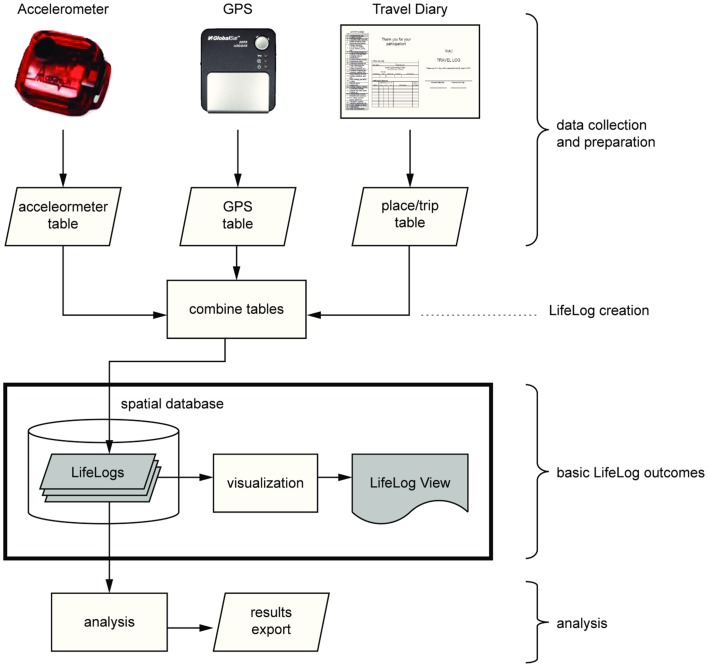
**Illustration of LifeLog work flow**.

Common timestamps are the “glue” that enables our three basic datasets (accelerometry, GPS, and travel diary) to come together. Each record from each data source is stored with an explicit timestamp, and tabular joins are enforced by common timestamps or time ranges across tables known as LifeLogs. A graphical work flow to create LifeLogs is shown in Figure 8; the basic SQL code for creating a LifeLog is provided in Example 1 in Supplementary Material.

Although creating LifeLogs from individual constituent tables is simple in PostgreSQL, some issues need to be addressed to ensure that tabular relationships are sound (e.g., all devices must have their “clocks” aligned).

##### Time zones

Each moment in time can be represented as a timestamp. Timestamps can be rounded to the nearest second with no loss of information to provide the level of precision needed in this type of spatial epidemiologic research. A commonly accepted standard is the number of seconds elapsed since January 1, 1970, 00:00 UTC (Coordinated Universal Time, or Greenwich time zone); this is often called “Unix time.” Several factors can introduce errors in timestamps. Although one of the benefits of UTC is that each moment can be represented unambiguously, errors result if time zones are not explicitly specified and handled. The R script shown in Example 2 in Supplementary Material illustrates how a timestamp can be handled to account for specific time zones. PostgreSQL has similar functionality.

When datasets containing timestamps are passed from one software package to another, careful attention is required to avoid errors resulting from conversions that assume that timestamps are stored in local time.

##### Daylight saving time

Across the U.S. and in many regions worldwide, daylight saving time is used to increase the number of daylight hours after the work day in summer. When clocks are set to change (“spring ahead” or “fall back”), they either lose or gain an hour. Unless completely specified timestamps are used with software that properly handles daylight saving time, errors are possible in measuring intervals that span the moment when daylight saving time begins or ends. The software packages used for LifeLog data processing and storage, R and PostgreSQL, correctly account for daylight savings time transitions as shown in Example 3 in Supplementary Material, but other software may not.

##### Analytic boundary for days

Midnight marks the transition between calendar days, but many people are active past midnight. In order to assign periods of activity to a behaviorally based unit, we decided to use 03:00 a.m. as the transition between analytic days. Any activity occurring between 23:59 and 02:59 was assigned to the previous calendar day. The simulated example in Example 4 in Supplementary Material shows the day transition after 02:50.

##### Timestamp rounding

Accelerometry timestamps are typically collected at regular intervals, such as 10, 15, 30, or 60 s. The GPS units are also configured to record at set intervals, but the actual time of acquisition is often more sporadic, depending on when the GPS unit can obtain a satellite fix. Therefore, to relate accelerometry records with GPS records, the records that are the closest in time in each dataset should be matched. One approach to matching is to loop through the accelerometry records and find the GPS record with the closest timestamp; however, this method is inefficient from a processing perspective. A better approach is first to determine the interval of the accelerometry recording and then to round the GPS timestamps to the same interval. Because several different GPS timestamps might round to the same value (e.g., 00:01 and 00:02 both round to 00:00), the GPS table is truncated to include records with unique rounded timestamps. Truncation should give precedence to the GPS timestamp closest to the rounded timestamp and delete other candidate matches (e.g., matching 00:00 with candidates 00:01 and 00:02 would retain 00:01 and delete 00:02). For ties (e.g., 00:01 and 00:59), PostgreSQL will select the first matching record in internal tabular order.

The SQL code for generating rounded timestamps shown in Example 5 in Supplementary Material allows the use of any interval. An example of the rounding function is also shown in Example 6 in Supplementary Material, which is based on a single participant’s data with a subset of results shown in Table 1. The raw GPS dataset for this study participant consisted of 29,382 records, but after rounding and selecting unique timestamp-rounded records, the resulting table contained 17,073 records. For a table of this size, the processing time was <1 s on a RedHat Linux machine with a 64-bit Intel Xeon E31270 3.40 GHz processor and 16 GB of RAM.

**Table 1:** **Illustration of original and rounded timestamps from one dataset**.

Rec.	Time_gps_utc	Time_gps_utc_std	Diff. time
1	2009-01-16 02:22:52	2009-01-16 02:23:00	−8
2	2009-01-16 02:23:22	2009-01-16 02:23:30	−8
3	2009-01-16 02:23:52	2009-01-16 02:24:00	−8
4	2009-01-16 02:24:22	2009-01-16 02:24:30	−8
5	2009-01-16 02:24:52	2009-01-16 02:25:00	−8
6	2009-01-16 02:25:22	2009-01-16 02:25:30	−8
7	2009-01-16 02:25:52	2009-01-16 02:26:00	−8
8	2009-01-16 02:26:22	2009-01-16 02:26:30	−8
9	2009-01-16 02:26:52	2009-01-16 02:27:00	−8
10	2009-01-16 02:27:22	2009-01-16 02:27:30	−8
11	2009-01-16 02:27:52	2009-01-16 02:28:00	−8
12	2009-01-16 02:28:35	2009-01-16 02:28:30	5
13	2009-01-16 08:44:33	2009-01-16 08:44:30	3
14	2009-01-16 08:45:06	2009-01-16 08:45:00	6
15	2009-01-16 08:45:39	2009-01-16 08:45:30	9
16	2009-01-16 08:46:12	2009-01-16 08:46:00	12
17	2009-01-16 08:46:45	2009-01-16 08:47:00	−15
18	2009-01-16 08:47:18	2009-01-16 08:47:30	−12
19	2009-01-16 08:47:51	2009-01-16 08:48:00	−9
20	2009-01-16 08:48:24	2009-01-16 08:48:30	−6

*Columns represent sequential record number, global positioning systems (GPS) measurement time in UTC (Coordinated Universal Time), GPS measurement time rounded to a standard 30 s interval, and the difference between raw and standardized timestamps*.

#### LifeLog View

**Figure 9:**
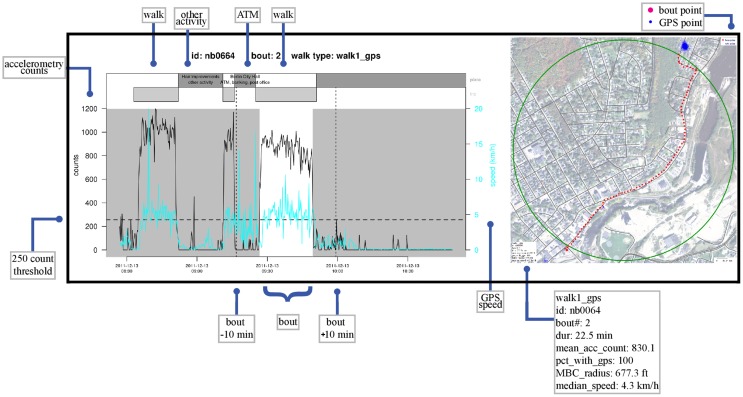
**An illustration of LifeLog View for a single bout of activity for one subject**. Left panel: time-indexed combined accelerometry counts (black lines), global positioning system (GPS) speed (cyan lines), and place and trip data from a travel diary (gray boxes with text labels). A physical activity bout is centered in the graph within a white background. The dashed horizontal line indicates the accelerometry threshold used to define a bout of walking. The dashed vertical lines are at 10 min on either side of the bout. Right panel: map of bout location. Larger red dots are individual GPS locations for the bout, with all GPS locations shown as small blue dots. The green circle indicates the boundary for the 95% most tightly clustered points in the bout.

**Figure 10:**
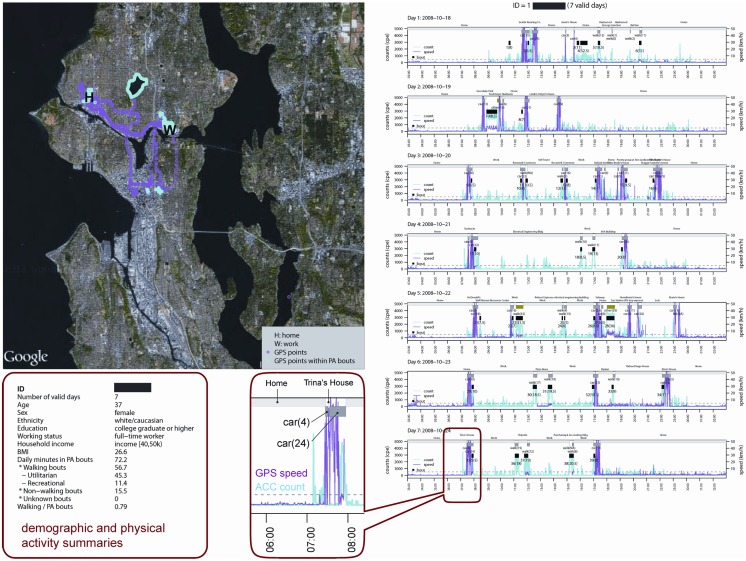
**Example LifeLog data collected over 1 week for a single subject**. Graphs (right) indicate accelerometry counts in cyan, GPS speed in magenta, places and trips (gray boxes), and physical activity bouts (black rectangles) for each day. The map (left) shows all GPS points in magenta and those that occurred within a physical activity bout in cyan, as well as home and work locations (“H” and “W” markers). A summary of demographic characteristics and overall subject-level activity, as well as a close-up of graph data, are shown in the callouts.

LifeLog Views provide multiple illustrations of complex data derived from the LifeLog. Data for a walking bout are illustrated in Figure 9. The left panel shows accelerometry, GPS, and place and trip information within the same temporal *X*-axis graph, created using R. The map portion of the LifeLog View (right panel) shows all GPS locations for a given study participant, with bout-specific GPS locations in red, and was created using University of Minnesota MapServer software ^1^. A green line identifies the minimum bounding circle drawn around 95% of the most tightly clustered points in the bout. Participant ID, sequential bout number, and activity type are printed as the main title of the image (top left). Each component image (graph and map) was created using automated scripts, and the images were automatically mosaicked using Imagemagick ^2^. Each data element in LifeLog View is useful for developing empirically based tolerances for activity classification. A second LifeLog View shows one combined accelerometry/GPS/diary graph per day (right panel) and GPS locations (left panel) collected over 1 week (Figure 10). LifeLog Views were instrumental in developing and validating the algorithms used to classify bouts of walking (32).

#### Analytical tools

Data compiled as LifeLogs can be used for many purposes. Because LifeLogs contain original data from all sources (accelerometry, GPS, and travel diary), they can be used for analyzing, graphing, and mapping of activities and locations, either as separate or combined components, as a spatially and temporally explicit database. Possible analyses are briefly discussed using the identification of physical activity and walking bouts as examples. Also presented is a new tool to graph and map all data in the LifeLog.

##### Physical activity bouts from accelerometry

Accelerometry data can be processed by using established methods to stratify records by levels of physical activity. We have considered periods of at least 20 min of zero accelerometry counts as non-wearing, while days with at least 8 h of wearing time are considered valid (33, 34). Within valid days, wearing and non-wearing intervals are differentiated following the approach described by Matthews and colleagues (35); intervals of at least 60 min of zero counts, with no more than two consecutive minutes of 1–50 counts per epoch (using a 30-s epoch), are coded as non-wearing. Sustained bouts of physical activity are defined as having accelerometry epochs above a threshold of 1000 counts per minute for at least 5 min, with allowance for 2 min of interstitial epochs below the threshold. A threshold of 1000 counts per minute is lower than the thresholds commonly used to represent moderate-to-vigorous physical activity (11) to identify walking bouts.

##### Classification of walking type from physical activity bouts

Processing accelerometry data alone allows us to identify bouts of physical activity and their relative intensity, but it provides no additional information on bout characteristics. Integrating GPS and travel diary data adds substantial power to contextualize physical activity bouts. GPS can characterize both the instantaneous speed and spatial clustering of individual locations within bouts. For example, place names, activity types, and transportation modes recorded in the travel diary can be used in conjunction with accelerometry and GPS data for fine-grained classification of walking types (32).

## Applications to Research

In this section, we describe two ongoing studies in which we apply our suite of tools to spatially oriented research questions. One study involves a natural experiment to evaluate the effects of new transportation system on physical activity levels. The other study evaluates neighborhood effects on physical activity, using identical twins as quasi-causal controls to overcome the self-selection and reverse causation problems that plague the literature on this topic.

### Travel assessment and community

The travel assessment and community (TRAC) study focuses on public transit use. Public transit users tend to engage in higher levels of physical activity than non-users. However, we want to know if users’ physical activity is directly attributable to transit use and/or changes in transit access. To address these questions, we need data that can tell us when study participants use transit and what kind of behavior they exhibit before and after transit trips. We hypothesize that they will walk to and from the points where they access public transit. Therefore, we need to determine whether physical activity that happens in the temporal vicinity of transit trips is consistent with walking or with some other form of activity, such as working out at a gym. Based on the methods described here, and reported by us recently (32), we have successfully used the LifeLog to identify the time, place, and type of physical activity performed for participants in the TRAC study, and to make estimates of the amount of physical activity directly attributable to transit use as described by us in a paper currently in press (36).

In the first longitudinal measurement phase in the TRAC study, we recruited 748 participants who had recorded data for any of the three instruments. Compliance with measures completion was relatively high; 715 participants had at least some data from each of the three instruments. Of the 701 participants with accelerometer and GPS data on valid days, there was a mean of 12.3 accelerometer wearing hours per day (SD, 1.6 h) and 11.3 GPS hours (SD, 7.3 h). The average accelerometer wear hours was slightly lower than reported in several other studies (between 12.5 and 14.2 h per day) (7, 8, 37); however, GPS wear times were not usually reported. Some participants who did not satisfactorily complete data collection were asked to re-wear the devices and fill out travel diaries for additional days; accelerometer data were collected from 730 participants, with 49 participants (6.7%) providing re-wear accelerometry data.

### TWIN study of environment, lifestyle behaviors, and health

Since 2008, all residential addresses for adult twins who are members of the University of Washington Twin Registry (UWTR) have been stored in a central database to enable temporal and spatial matching with survey data. The Registry is now poised to take advantage of the array of data assembled over the past several years in analyses of associations among genetic, environmental, behavioral, and health variables. Such analyses depend on linking all our available data types (survey, biological, and environmental). Because twin participants in the Registry are surveyed every 2 years, we are also able to follow them longitudinally to investigate temporal associations between changes in built and social environments and changes in activity behaviors.

Each individual twin’s home address is geocoded in ArcGIS by using ESRI (Redlands, CA, USA) StreetMap Premium with a minimum match score of 100%. Addresses that fail the automatic geocoding process (~40%) are matched manually. The following are examples of the environmental exposures we use in our research: neighborhood walkability (22, 27, 38–44), level of urban sprawl (45), amount of vegetation or “green space” (46, 47), material and social deprivation (48), residential property values (49, 50), and crime rates (51). These indices rely on multiple data sources, including the U.S. Census, parcel-level and tax-lot level data, county-level assessor data, and InfoUSA, a commercially available resource that provides information on food sources as well as fitness, service, and retail facilities. Point-in-polygon analysis attaches values from our environmental indices to each twin by using the twin’s geocoded residential address. Although much of our environmentally based work focused on the residential neighborhood, newer studies such as the one described in the paragraph below also include data on the work and school environment, as well as “distal” environments that participants might frequent on a regular basis (e.g., a favorite coffee shop, a gym, etc.). Thanks to the novel applications on which we focus in this article, we can now exploit the full activity space over time.

**Figure 11:**
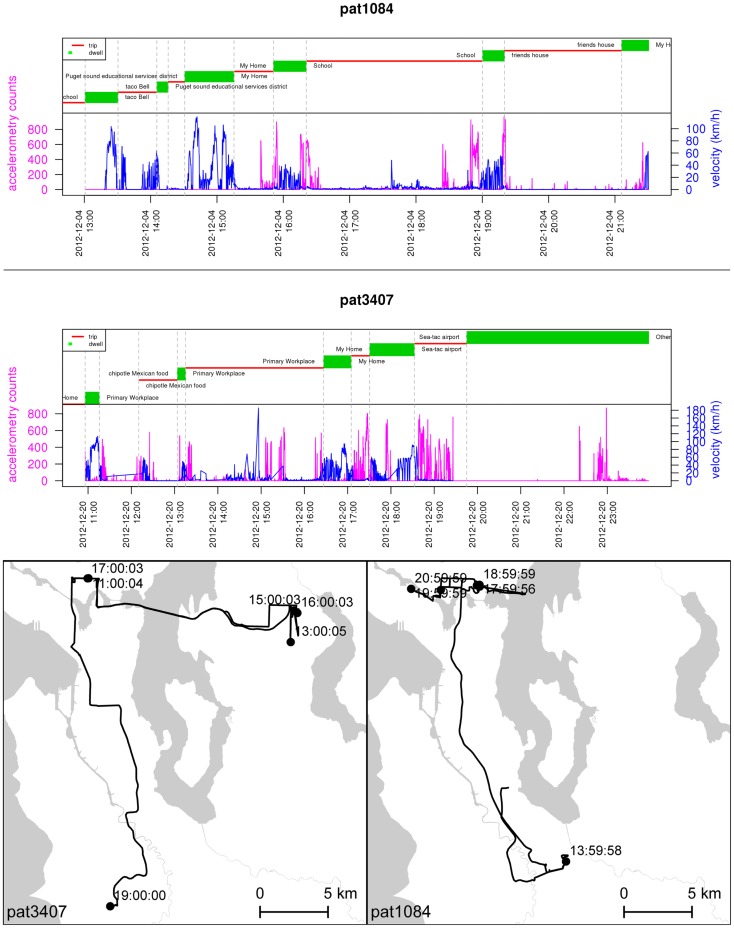
**One-day LifeLogs collected from a twin pair**. The upper panel shows self-reported data on place (red) and trip (green) from the travel diary along with objective accelerometry counts (magenta) and GPS velocity (blue). The lower panel maps travel patterns for the same individuals over the same days, with markers shown at hourly intervals.

In this research, we will investigate the effects of the built environment on lifestyle behaviors and health in a community-based sample of 200 adult monozygotic twin pairs (400 individuals) from the UWTR who were reared together but now live apart. This unique sample will permit us to examine environmental influences on lifestyle behaviors and health, free of the genetic and shared environmental (familial) effects that might otherwise introduce selection biases into the choice of living environments. We describe each twin’s residential environment in terms of the indices previously noted. Participants are outfitted with an accelerometer, a GPS data logger, and an Android smartphone for continuous tracking in time and space over 2 weeks. The data from these three tools are joined in a LifeLog indexed by common timestamps across devices. An example of data collected for one twin pair is shown in Figure 11; LifeLog data will assist us in investigating multiple issues. For example, we will determine the association between the home-built environment and levels of both walking and total physical activity in twins who live apart. We will also compare location-based physical activity and eating episodes in real-time to assess whether proximity to features of the home-built environment are associated with use by measuring how many physical activity and eating episodes occur in the home-built environment versus in-distal built environments, including work, transit, and recreation-related settings. Our study design is notable in several ways: it overcomes the measurement bias inherent in self-report data, addresses the problem of defining “neighborhood,” and engages in novel spatial–temporal measures of behaviors that correspond to ecological exposures.

To date, we have completed data collection on 70 twin pairs. Compliance with wearing the devices has been exceptional; of 106 individual twins whose data has been processed thus far, average wearing days for the accelerometer and GPS is 13.8 (out of 14 days of measurement). The average wearing days for the mobile phone with entries for *MyFootprints* and *Travel Diary* are 6.6 and 7 days, respectively (out of 7 days of measurement for each program). Of course, when we enter the data analysis phase our group will need to determine the actual number of valid wear days based on the number of valid hours for each day for each device. Nonetheless, this preliminary “peak” at the data on wear time is promising.

## Limitations

Our methods and analyses are based on objective measures of location and physical activity; however, the devices and basic data processing methods for these are not perfect. GPS data of sufficient duration and quality are challenging given such inherent problems as urban canyons, cold starts, and limited battery life. When GPS data are not present, it is not possible to determine whether data loss was due to power being turned off or loss of signal. Likewise, when a GPS is powered on and recording, but not worn (e.g., recording when charging overnight), data will be logged even though these will not reflect actual movement patterns. Newer generation locational technologies using combined GPS and WiFi triangulation combined with other sensors for detecting movement through space are likely to provide better locational data in the near future.

Capturing behavior through time is problematic; we still rely on participants to record their travel and activity behavior. Although the use of smartphones as diary recording devices provides benefits such as obviating the need for data transcription and automatic time-stamping of recorded activities, there is still a relatively high participant burden to enter travel and activity information, regardless of the instrument used. Several investigators are exploring the use of portable cameras to capture periodic images for use in activity classification (52–54); however, such methods rely on manual annotation of images, which is a tedious and lengthy process. At this time, it is unknown when a reliable method for automatically classifying behaviorally defined activity types will be developed.

## Conclusion

In summary, there is a growing interest in obtaining more precision and more information about the amount, type, and context of physical activity and other health behaviors. Newer devices (e.g., portable GPS) and their combined use offer opportunities to improve precision and collect this additional information. However, standard methods and procedures are needed to best capture and integrate the large volume of data obtained from these devices. The integrative characteristics of the large datasets contained in LifeLogs and SmartMaps hold great promise for advancing spatial epidemiologic research, especially work whose goal is to facilitate behaviors that promote health.

## Conflict of Interest Statement

The authors declare that the research was conducted in the absence of any commercial or financial relationships that could be construed as a potential conflict of interest.

## Supplementary Material

The Supplementary Material for this article can be found online at http://www.frontiersin.org/Journal/10.3389/fpubh.2014.00002/abstract

## Footnotes

^1^http://mapserver.org/

^2^http://www.imagemagick.org

## References

[1] Helmrich SP Ragland DR Leung RW Paffenbarger RS Jr. Physical activity and reduced occurrence of non-insulin-dependent diabetes mellitus. N Engl J Med (1991) 325(3):147–52 10.1056/NEJM199107183250302 2052059

[2] Knowler WC Barrett-Connor E Fowler SE Hamman RF Lachin JM Walker EA Reduction in the incidence of type 2 diabetes with lifestyle intervention or metformin. N Engl J Med (2002) 346(6):393–403 10.1056/NEJMoa012512 11832527 PMC1370926

[3] Leon AS Myers MJ Connett J Leisure time physical activity and the 16-year risks of mortality from coronary heart disease and all-causes in the multiple risk factor intervention trial (MRFIT). Int J Sports Med (1997) 18(Suppl 3):S208–15 10.1055/s-2007-972717 9272851

[4] Wannamethee SG Shaper AG Physical activity in the prevention of cardiovascular disease: an epidemiological perspective. Sports Med (2001) 31(2):101–14 10.2165/00007256-200131020-00003 11227978

[5] Vainio H Kaaks R Bianchini F Weight control and physical activity in cancer prevention: international evaluation of the evidence. Eur J Cancer Prev (2002) 11(Suppl 2):S94–100 12570341

[6] Centers for Disease Control and Prevention Adult participation in recommended levels of physical activity – United States, 2001 and 2003. MMWR Morb Mortal Wkly Rep (2005) 54(47):1208–12 16319815

[7] Troiano RP Berrigan D Dodd KW Masse LC Tilert T McDowell M Physical activity in the United States measured by accelerometer. Med Sci Sports Exerc (2008) 40(1):181–8 10.1249/mss.0b013e31815a51b3 18091006

[8] Rodriguez DA Cho GH Evenson KR Conway TL Cohen D Ghosh-Dastidar B Out and about: association of the built environment with physical activity behaviors of adolescent females. Health Place (2012) 18(1):55–62 10.1016/j.healthplace.2011.08.020 21945085 PMC3259163

[9] The PostgreSQL Global Development Group. *PostgreSQL* Available from: http://www.postgresql.org/

[10] The R Development Core Team R: A Language and Environment for Statistical Computing Reference Index (ver 2.11.1). Heidelberg: University of Heidelberg (2012). 1651 p. Available from: http://www.lsw.uni-heidelberg.de/users/christlieb/teaching/UKStaSS10/R-refman.pdf

[11] Freedson PS Melanson E Sirard J Calibration of the Computer Science and Applications, Inc. accelerometer. Med Sci Sports Exerc (1998) 30:777–81 10.1097/00005768-199805000-00021 9588623

[12] Pober DM Staudenmayer J Raphael C Freedson PS Development of novel techniques to classify physical activity mode using accelerometers. Med Sci Sports Exerc (2006) 38(9):1626–34 10.1249/01.mss.0000227542.43669.45 16960524

[13] Freedson PS Lyden K Kozey-Keadle S Staudenmayer J Evaluation of artificial neural network algorithms for predicting METs and activity type from accelerometer data: validation on an independent sample. J Appl Physiol (2011) 111(6):1804–12 10.1152/japplphysiol.00309.2011 21885802 PMC3233887

[14] Lyden K Keadle SK Staudenmayer J Freedson PS A method to estimate free-living active and sedentary behavior from an accelerometer. Med Sci Sports Exerc (2013) 46(2):386–97 10.1249/MSS.0b013e3182a42a2d 23860415 PMC4527685

[15] The PostGIS Development Group TPD. *PostGIS* Available from: http://postgis.net/

[16] Lester J Choudhury T Kern N Borriello G Hannaford B A Hybrid Discriminative/Generative Approach for Modeling Human Activities. Proceedings of the 19th International Joint Conference on Artificial Intelligence Edinburgh: Morgan Kaufmann Publishers, Inc. (2005). p. 766–72

[17] Duncan GE Lester J Migotsky S Goh J Higgins L Borriello G Accuracy of a novel multi-sensor board for measuring physical activity and energy expenditure. Eur J Appl Physiol (2011) 111(9):2025–32 10.1007/s00421-011-1834-2 21249383 PMC3124601

[18] Duncan GE Lester J Migotsky S Higgins L Borriello G Measuring slope to improve energy expenditure estimates during field-based activities. Appl Physiol Nutr Metab (2013) 38(3):352–6 10.1139/apnm-2012-0223 23537030 PMC3753037

[19] Lee C Moudon AV The 3Ds+R: quantifying land use and urban form correlates of walking. Trans Res Part D-Trans Environ (2006) 11(3):204–15 10.1016/j.trd.2006.02.003

[20] Rushton G Public health, GIS and spatial analytic tools. Annu Rev Public Health (2003) 24:43–56 10.1146/annurev.publhealth.24.012902.140843 12471269

[21] Moudon AV Sohn DW Kavage S Mabry JE Transportation-efficient land use mapping index (TELUMI), a tool to assess multimodal transportation options in metropolitan regions. Int J Sustain Trans (2011) 5:111–33 10.1080/15568311003624262

[22] Moudon AV Lee C Cheadle AD Garvin C Rd DB Schmid TL Attributes of environments supporting walking. Am J Health Promot (2007) 21(5):448–59 10.4278/0890-1171-21.5.448 17515010

[23] Badland HM Schofield GM Garrett N Travel behavior and objectively measured urban design variables: associations for adults traveling to work. Health Place (2008) 14:85–95 10.1016/j.healthplace.2007.05.002 17590378

[24] McConville ME Rodriguez DA Clifton K Cho G Fleischhacker S Disaggregate land uses and walking. Am J Prev Med (2011) 40(1):25–32 10.1016/j.amepre.2010.09.023 21146764

[25] McCormack GR Giles-Corti B Bulsara M The relationship between destination proximity, destination mix and physical activity behaviors. Prev Med (2008) 46(1):33–40 10.1016/j.ypmed.2007.01.013 17481721

[26] McGinn AP Evenson KR Herring AH Huston SL Rodríguez DA Exploring associations between physical activity and perceived and objective measures of the built environment. J Urban Health (2007) 84(2):162–84 10.1007/s11524-006-9136-4 17273926 PMC2231636

[27] Frank LD Saelens BE Powell KE Chapman JE Stepping towards causation: do built environments or neighborhood and travel preferences explain physical activity, driving, and obesity? Soc Sci Med (2007) 65(9):1898–914 10.1016/j.socscimed.2007.05.053 17644231

[28] Fitzhugh EC Bassett DR Jr. Evans MF Urban trails and physical activity: a natural experiment. Am J Prev Med (2010) 39(3):259–62 10.1016/j.amepre.2010.05.010 20709258

[29] Jiao J Moudon AV Drewnowski A Grocery shopping: how individuals and built environments influence travel mode choice. Trans Res Rec (2011) 2230:85–95 10.3141/2230-10 PMC4341903 25729127

[30] Hurvitz PM Moudon AV Home versus nonhome neighborhood: quantifying differences in exposure to the built environment. Am J Prev Med (2012) 42(4):411–7 10.1016/j.amepre.2011.11.015 22424255 PMC3318915

[31] de Smith MJ Goodchild MF Longley PA Geospatial Analysis: A Comprehensive Guide to Principles, Techniques and Software Tools. 3rd ed Leicester: Troubadour Publishing, Ltd (2009). 394 p.

[32] Kang B Moudon AV Hurvitz PM Reichley L Saelens BE Walking objectively measured: classifying accelerometer data with GPS and travel diaries. Med Sci Sports Exerc (2013) 45(7):1419–28 10.1249/MSS.0b013e318285f202 23439414 PMC3674121

[33] Masse LC Fuemmeler BF Anderson CB Matthews CE Trost SG Catellier DJ Accelerometer data reduction: a comparison of four reduction algorithms on select outcome variables. Med Sci Sports Exerc (2005) 37:S544–54 10.1249/01.mss.0000185674.09066.8a 16294117

[34] Reilly JJ Kelly La Montgomery C Jackson DM Slater C Grant S Validation of actigraph accelerometer estimates of total energy expenditure in young children. Int J Pediatr Obes (2006) 1:161–7 10.1080/17477160600845051 17899634

[35] Matthews CE Chen KY Freedson PS Buchowski MS Beech BM Pate RR Amount of time spent in sedentary behaviors in the United States, 2003–2004. Am J Epidemiol (2008) 167(7):875–81 10.1093/aje/kwm390 18303006 PMC3527832

[36] Saelens BE Moudon AV Kang B Hurvitz PM Zhou C Higher physical activity is directly related to public transit use. Am J Public Health (Forthcoming). 10.2105/AJPH.2013.301696 PMC3987609 24625142

[37] Hagstromer M Oja P Sjostrom M Physical activity and inactivity in an adult population assessed by accelerometry. Med Sci Sports Exerc (2007) 39(9):1502–8 10.1249/mss.0b013e3180a76de5 17805081

[38] Lee C Moudon AV Correlates of walking for transportation or recreation purposes. J Phys Act Health (2006) 3:S77–98 10.1123/jpah.3.s1.s77 28834524

[39] Moudon AV Lee C Cheadle AD Garvin C Johnson D Schmid TL Operational definitions of walkable neighborhood: theoretical and empirical insights. J Phys Act Health (2006) 3:S99–117 10.1123/jpah.3.s1.s99 28834523

[40] Frank LD Schmid TL Sallis JF Chapman J Saelens BE Linking objectively measured physical activity with objectively measured urban form: findings from SMARTRAQ. Am J Prev Med (2005) 28(2 Suppl 2):117–25 10.1016/j.amepre.2004.11.001 15694519

[41] Leslie E Coffee N Frank L Owen N Bauman A Hugo G Walkability of local communities: using geographic information systems to objectively assess relevant environmental attributes. Health Place (2007) 13(1):111–22 10.1016/j.healthplace.2005.11.001 16387522

[42] Walk Score. *Walk Score Methodology* (2011). Available from: http://www.walkscore.com/methodology.shtml

[43] Carr LJ Dunsiger SI Marcus BH Walk score (TM) as a global estimate of neighborhood walkability. Am J Prev Med (2010) 39(5):460–3 10.1016/j.amepre.2010.07.007 20965384 PMC4845902

[44] Carr LJ Dunsiger SI Marcus BH Validation of walk score for estimating access to walkable amenities. Br J Sports Med (2011) 45(14):1144–8 10.1136/bjsm.2009.069609 20418525 PMC4845899

[45] Ewing R Schmid T Killingsworth R Zlot A Raudenbush S Relationship between urban sprawl and physical activity, obesity, and morbidity. Am J Health Promot (2003) 18(1):47–57 10.4278/0890-1171-18.1.47 13677962

[46] Rhew IC Vander Stoep A Kearney A Smith NL Dunbar MD Validation of the normalized difference vegetation index as a measure of neighborhood greenness. Ann Epidemiol (2011) 21(12):946–52 10.1016/j.annepidem.2011.09.001 21982129 PMC3225119

[47] Tilt JH Unfried TM Roca B Using objective and subjective measures of neighborhood greenness and accessible destinations for understanding walking trips and BMI in Seattle, Washington. Am J Health Promot (2007) 21(4):371–9 10.4278/0890-1171-21.4s.371 17465183

[48] Singh GK Area deprivation and widening inequalities in US mortality, 1969–1998. Am J Public Health (2003) 93(7):1137–43 10.2105/AJPH.93.7.1137 12835199 PMC1447923

[49] Rehm CD Moudon AV Hurvitz PM Drewnowski A Residential property values are associated with obesity among women in King County, WA, USA. Soc Sci Med (2012) 75(3):491–5 10.1016/j.socscimed.2012.03.041 22591823 PMC3889661

[50] Moudon AV Cook AJ Ulmer J Hurvitz PM Drewnowski A A neighborhood wealth metric for use in health studies. Am J Prev Med (2011) 41(1):88–97 10.1016/j.amepre.2011.03.009 21665069 PMC3118096

[51] Doyle S Kelly-Schwartz A Schlossberg M Stockard J Active community environments and health – the relationship of walkable and safe communities to individual health. J Am Plann Assoc (2006) 72(1):19–31 10.1080/01944360608976721

[52] Doherty AR Kelly P Kerr J Marshall S Oliver M Badland HM Using wearable cameras to categorise type and context of accelerometer-identified episodes of physical activity. Int J Behav Nutr Phys Act (2013) 10:22 10.1186/1479-5868-10-22 23406270 PMC3615956

[53] Kerr J Marshall SJ Godbole S Chen J Legge A Doherty AR Using the sensecam to improve classifications of sedentary behavior in free-living settings. Am J Prev Med (2013) 44:290–6 10.1016/j.amepre.2012.11.004 23415127

[54] Gurrin C Qiu Z Hughes M Caprani N Doherty AR Hodges SE The smartphone as a platform for wearable cameras in health research. Am J Prev Med (2013) 44:308–13 10.1016/j.amepre.2012.11.010 23415130

