# Supplementary material for: Emerging Technologies for Assessing Physical Activity Behaviors in Space and Time
Source: Front Public Health. 2014 Jan 28;2:2. doi: 10.3389/fpubh.2014.00002 (PMC3904281; doi:10.3389/fpubh.2014.00002)
Supplement: Supplementary file 1 [file 75633_Hurvitz_DataSheet1.DOCX]

**Supplementary material**

The supplementary material includes example code and syntax for data integration and management operations. Explanations are presented below code boxes; code examples are separated by horizontal border lines.

Example 1. Creating a LifeLog from accelerometry, GPS, and travel diary tables.

| 1 SELECT DISTINCT on (time_acc_utc) *  2 FROM acc_wa001 AS a  3 FULL JOIN trips AS t  4 ON a.id = t.id  5 AND COALESCE(a.time_acc_utc <= t.time_end_utc, a.time_acc_utc <= t.time_start_utc)  6 AND COALESCE(a.time_acc_utc >= t.time_start_utc, a.time_acc_utc >= t.time_end_utc)  7 FULL JOIN places AS p  8 ON a.id = p.id  9 AND COALESCE(a.time_acc_utc >= p.time_arrived_utc, a.time_acc_utc >= p.time_left_utc)  10 AND COALESCE(a.time_acc_utc <= p.time_left_utc, a.time_acc_utc <= p.time_arrived_utc)  11 FULL JOIN (  12 SELECT DISTINCT ON (time_gps_utc_std) *  13 FROM gps_wa001  14 ) AS g ON a.id = g.id  15 AND a.time_acc_utc = g.time_gps_utc_std  16 WHERE a.id IS NOT NULL  17 ORDER BY a.time_acc_utc  18 , recnum_place |
| --- |

Example SQL code for creating LifeLogs, using subject ID “wa001” as an example. Fields and tables are specified as: “time_acc_utc” are UTC timestamps from the accelerometry table (“acc_wa001”); “time_start_utc” and “time_end_utc” are start and end times for travel log trips (“trips”); time_arrived_utc” and “time_left_utc” are arrival and departure times for travel log places (“places”); “time_gps_utc_std” is the rounded timestamp from the GPS table (“gps_wa001”); “recnum_place” is the sequential place number from the travel log.

Lines 1-2 select a set of records that are unique with respect to accelerometry timestamp.

Lines 3-6 joins trips data based on timestamp overlaps between accelerometry and trips.

Lines 7-10 joins places data, also based on timestamp overlaps.

Lines 11-15 select uniquely timestamped GPS records and then joins to accelerometry using standardized GPS timestamp.

Lines 16-18 select only records where there was a value for the “id” field, and order by accelerometry timestamp and place number.

Example 2. How a POSIX timestamp values are assigned to time zones in R.

| 1 (A = Sys.time())  2 [1] "2013-10-16 14:03:43 PDT"  3 attr(A, "tzone") = "UTC"  4 A  5 [1] "2013-10-16 21:03:43 UTC"  6 as.POSIXct("2013-10-16 14:03:43")  7 [1] "2013-10-16 14:03:43 PDT"  8 as.POSIXct("2013-10-16 14:03:43", tz="UTC")  9 [1] "2013-10-16 14:03:43 UTC" |
| --- |

Line 1 assigns the current system time to variable *A*.

Line 2 shows that the timestamp is automatically set to local time (PDT).

Line 3 assigns Coordinated Universal Time to the timestamp stored in variable *A*.

Lines 4-5 print the value of *A*, showing time conversion.

Lines 6-7 show how a timestamp that is created without a time zone specified defaults to local time.

Lines 8-9 show how to explicitly set a time zone on a created timestamp.

Example 3. How daylight savings time is handled in R.

| > a = as.POSIXct("2013-03-10 01:59")  > data.frame(spring_ahead=a,"minus_1_hr"=a-3600, plus_1_hr=a+3600)  spring_ahead minus_1_hr plus_1_hr  1 2013-03-10 01:59:00 2013-03-10 00:59:00 2013-03-10 03:59:00 |
| --- |

Variable *a* is assigned to a timestamp just before the change from daylight savings to standard time (01:59 AM on Mar 10, 2013). Sixty minutes are added/subtracted, resulting in respective timestamps of 00:59 AM and 03:59 AM.

Example 4. Assigning analytic days (breaks at 3 AM).

| **1 > require(data.table)**  **2 > x=as.POSIXct("2013-10-16 23:58:30")**  **3 > Y=data.frame(tstamp=seq(x, x+3600*4.5, by="60 secs"))**  **4 > Y$jday_adj=yday(Y$tstamp - 3*3600)**  **5 > Y**  tstamp jday_adj  1 2013-10-16 23:58:30 289  2 2013-10-16 23:59:30 289  3 2013-10-17 00:00:30 289  ...  181 2013-10-17 02:58:30 289  182 2013-10-17 02:59:30 289  183 2013-10-17 03:00:30 290  184 2013-10-17 03:01:30 290  185 2013-10-17 03:02:30 290 |
| --- |

Code is presented in **bold**, above the tabular output.

Line 1 loads the “data.table” library into the current R session.

Line 2 creates a timestamp variable *x* as 2013-10-16 23:58:30.

Line 3 creates a data frame, adding 4.5 hours of records at a 60 s interval.

Line 4 creates the “jday_adj” column as the Julian day number applied to timestamps shifted by 3 hours.

The output shows for lines 1-182 (from 23:58:30 to 02:59:30) the Julian day of 289, with day 290 starting at 03:00.

Example 5. SQL function for generating rounded timestamps.

| CREATE OR REPLACE FUNCTION date_round(base_date timestamp with time zone,  round_interval interval) RETURNS timestamp with time zone AS  $BODY$  SELECT  TIMESTAMP WITH TIME ZONE 'epoch' + ( EXTRACT ( EPOCH  FROM  $1 ) ::INTEGER + EXTRACT ( EPOCH  FROM  $2 ) ::INTEGER / 2 )  / EXTRACT ( EPOCH  FROM  $2 ) ::INTEGER * EXTRACT ( EPOCH  FROM  $2 ) ::INTEGER * INTERVAL '1 second';  $BODY$  LANGUAGE sql STABLE  COST 100; |
| --- |

The function uses two arguments: timestamp and rounding interval, and returns a set of rounded timestamps.

Example 6. Timestamp rounding in PostgreSQL.

| **1 SELECT recnum_gps**  **2 , time_gps_utc**  **3 , time_gps_utc_std**  **4 , difftime**  **5 FROM (**  **6 SELECT ***  **7 , abs(extract(epoch FROM time_gps_utc - time_gps_utc_std)) AS difftime**  **8 FROM (**  **9 SELECT recnum_gps**  **10 , time_gps_utc**  **11 , *date_round(time_gps_utc, interval '30 seconds')::TIMESTAMP AS***  **12  *time_gps_utc_std***  **13 FROM gps_12410447**  **14 ) AS foo**  **15 ) AS foo**  **15 LIMIT 15;**  recnum_gps \| time_gps_utc \| time_gps_utc_std \| difftime  ------------+---------------------+---------------------+----------  1 \| 2009-01-16 02:22:38 \| 2009-01-16 02:22:30 \| 8  2 \| 2009-01-16 02:23:08 \| 2009-01-16 02:23:00 \| 8  3 \| 2009-01-16 02:23:38 \| 2009-01-16 02:23:30 \| 8  4 \| 2009-01-16 02:24:08 \| 2009-01-16 02:24:00 \| 8  5 \| 2009-01-16 02:24:38 \| 2009-01-16 02:24:30 \| 8  6 \| 2009-01-16 02:25:08 \| 2009-01-16 02:25:00 \| 8  7 \| 2009-01-16 02:25:38 \| 2009-01-16 02:25:30 \| 8  8 \| 2009-01-16 02:26:08 \| 2009-01-16 02:26:00 \| 8  9 \| 2009-01-16 02:26:38 \| 2009-01-16 02:26:30 \| 8  10 \| 2009-01-16 02:27:08 \| 2009-01-16 02:27:00 \| 8  11 \| 2009-01-16 02:27:38 \| 2009-01-16 02:27:30 \| 8  12 \| 2009-01-16 02:28:21 \| 2009-01-16 02:28:30 \| 9  13 \| 2009-01-16 08:44:19 \| 2009-01-16 08:44:30 \| 11  14 \| 2009-01-16 08:44:52 \| 2009-01-16 08:45:00 \| 8  15 \| 2009-01-16 08:45:25 \| 2009-01-16 08:45:30 \| 5 |
| --- |

In this example the timestamp field is *time_gps_utc* and the interval is *30 seconds*. Code is presented in **bold** font above the tabular results.

Lines 1-4 select fields from the subquery in lines 6-15.

Line 6 selects all columns from the subquery in lines 9-14.

Line 7 calculates a column “difftime” as the absolute difference in seconds between raw and rounded GPS timestamps.

Lines 9-10 select the sequential record number and raw timestamp from the GPS table.

Line 11 performs the timestamp rounding of 30 s on the GPS timestamp column.

Line 13 specifies the source table.

Line 15 limits to the first 15 returned records.
